# 3-(9*H*-Fluoren-9-yl)-3-(4-methyl­phen­yl)-1-phenyl­propan-1-one

**DOI:** 10.1107/S1600536812036847

**Published:** 2012-08-31

**Authors:** Wei-Bing Hu, Zhi-Cai Cui, Xin-Ping Liu, Fu Feng

**Affiliations:** a School of Chemical and Environmental Engineering, Hubei University for Nationalities, Enshi, Hubei 445000, People’s Republic of China

## Abstract

In the title compound, C_29_H_24_O, the phenyl and methyl­phenyl rings are approximately perpendicular to each other, making a dihedral angle of 87.67 (10)°, and are oriented at dihedral angles of 62.49 (9) and 84.77 (7)°, respectively, to the nearly planar fluorene ring system [maximum deviation = 0.077 (2) Å] In the crystal, weak C—H⋯π inter­actions are observed.

## Related literature

For the background to fluorene and its derivatives, see: Kreyenschmidt *et al.* (1998 ▶).
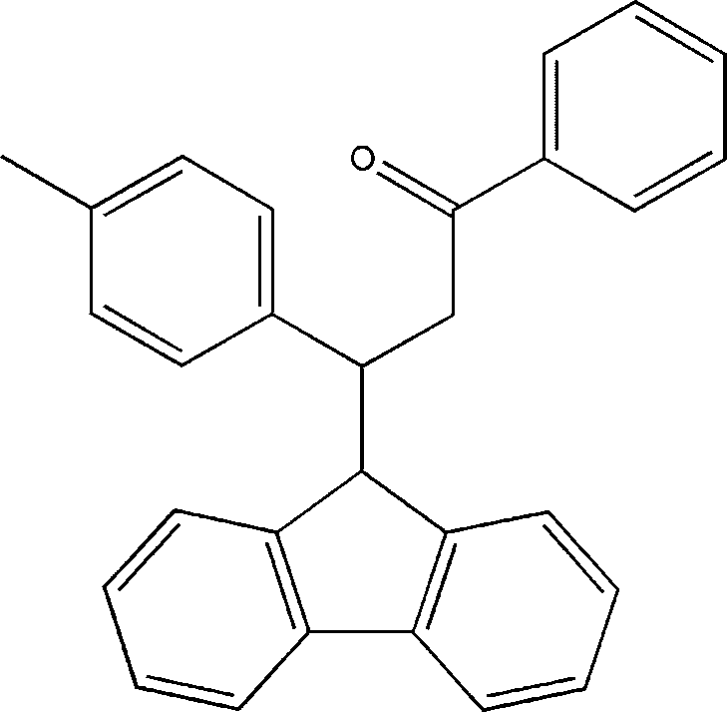


## Experimental

### 

#### Crystal data

C_29_H_24_O
*M* *_r_* = 388.48
Triclinic, 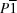

*a* = 9.7188 (16) Å
*b* = 10.8189 (18) Å
*c* = 11.5132 (19) Å
α = 75.500 (3)°
β = 68.681 (3)°
γ = 84.315 (3)°
*V* = 1091.8 (3) Å^3^
*Z* = 2
Mo *K*α radiation
μ = 0.07 mm^−1^
*T* = 298 K
0.26 × 0.20 × 0.10 mm

#### Data collection

Bruker SMART 1000 CCD area-detector diffractometer
11991 measured reflections
4508 independent reflections
3125 reflections with *I* > 2σ(*I*)
*R*_int_ = 0.022

#### Refinement

*R*[*F* ^2^ > 2σ(*F* ^2^)] = 0.049
*wR*(*F* ^2^) = 0.138
*S* = 1.07
4508 reflections
272 parameters
H-atom parameters constrained
Δρ_max_ = 0.16 e Å^−3^
Δρ_min_ = −0.14 e Å^−3^

### 

Data collection: *SMART* (Bruker, 2001 ▶); cell refinement: *SAINT* (Bruker, 2001 ▶); data reduction: *SAINT*; program(s) used to solve structure: *SHELXTL* (Sheldrick, 2008 ▶); program(s) used to refine structure: *SHELXTL*; molecular graphics: *SHELXTL*; software used to prepare material for publication: *SHELXTL*.

## Supplementary Material

Crystal structure: contains datablock(s) global, I. DOI: 10.1107/S1600536812036847/xu5614sup1.cif

Structure factors: contains datablock(s) I. DOI: 10.1107/S1600536812036847/xu5614Isup2.hkl

Supplementary material file. DOI: 10.1107/S1600536812036847/xu5614Isup3.cml

Additional supplementary materials:  crystallographic information; 3D view; checkCIF report

## Figures and Tables

**Table 1:** Hydrogen-bond geometry (Å, °) *Cg* is the centroid of the C10–C15 ring.

*D*—H⋯*A*	*D*—H	H⋯*A*	*D*⋯*A*	*D*—H⋯*A*
C27—H27⋯*Cg* ^i^	0.93	2.76	3.617 (2)	154

Symmetry code: (i) 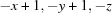.

**Fig. 1.**
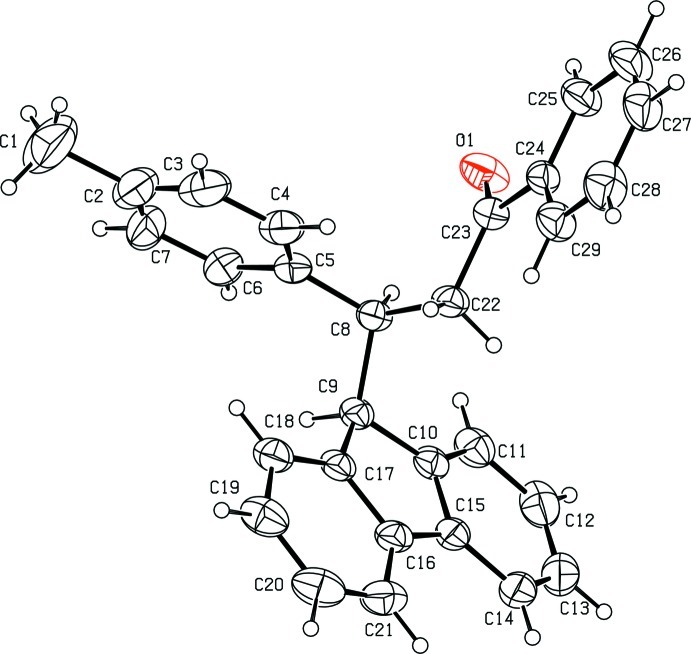
The molecular structure of (I), showing 50% probability displacement ellipsoids.

## supplementary crystallographic information

### Comment

Since the publication of its solid-state structure of fluorene and its derivatives are very useful compounds due to their good optical property and high luminescent efficiencies, and have received a lot of attentiont (Kreyenschmidt *et al.*, 1998). To our knowledge, a great number of studies have appeared on fluorene and its derivatives. The title compound (I) is an example of this class.

In the title compound, the phenyl ring and methylphenyl ring are approximately perpendicular to each other with a dihedral angle of 87.67 (10)°, and they are oriented with respect to the nearly planar fluorene ring systems [the maximum deviation being 0.077 (2) Å] at 62.49 (9) and 84.77 (7)°, respectively. In the crystal, weak intermolecular C—H···π interaction is observed (Table 1).

### Experimental

Fluorene (2 mmo1), chalcone (2 mmo1) and NaOH (4 mmo1) were mixed in mortar, and the mixture was ground at room temperature for 30 min. Then, the mixture was washed in sequence with 15 ml aqueous solution of HCl (3%) and alcohol (95%), and the crude product was isolated by filtration. The filtrate was purified by recrystallization from anhydrous ethanol to give compound I as colourless crystals in 73% yield. Suitable crystals for X-ray analysis were obtained by slow evaporation of a methanol solution at room temperature (m.p. 445–447 K); IR (KBr, ν cm^-1^): 3055, 3010, 2915, 1680, 1596, 1445, 1316, 1238, 813, 685; ^1^H NMR (DMSO-d_6_, δ): 6.83–8.07 (m, 17H); 3.97 (d, 1H, J = 4.2 Hz), 3.57 (m, 1H), 3.41 (d, 2H, J = 7.0 Hz), 2.19 (s, 3H); Elemental analysis calculated for C_29_H_24_O: C 86.69, H6.19%; found: C 86.81, H 6.12%.

### Refinement

All H atoms were placed in geometrically idealized positions and constrained to ride on their parent atoms, with C—H = 0.93 Å for phenyl H atoms, C—H = 0.96 Å for methyl H atoms, C—H = 0.97 Å for methylene H atoms, C—H = 0.98 Å for methylidyne H atoms and *U*_iso_(H) = 1.5*U*_eq_(C).

### Crystal data

**Table:**

C_29_H_24_O	*Z* = 2
*M**_r_* = 388.48	*F*(000) = 412
Triclinic, *P*1	*D*_x_ = 1.182 Mg m^−^^3^
Hall symbol: -P 1	Mo *K*α radiation, λ = 0.71073 Å
*a* = 9.7188 (16) Å	Cell parameters from 2741 reflections
*b* = 10.8189 (18) Å	θ = 2.3–24.1°
*c* = 11.5132 (19) Å	µ = 0.07 mm^−^^1^
α = 75.500 (3)°	*T* = 298 K
β = 68.681 (3)°	Block, colourless
γ = 84.315 (3)°	0.26 × 0.20 × 0.10 mm
*V* = 1091.8 (3) Å^3^	

### Data collection

**Table:**

Bruker SMART 1000 CCD area-detector diffractometer	3125 reflections with *I* > 2σ(*I*)
Radiation source: fine-focus sealed tube	*R*_int_ = 0.022
Graphite monochromator	θ_max_ = 26.5°, θ_min_ = 1.9°
phi and ω scans	*h* = −12→12
11991 measured reflections	*k* = −13→13
4508 independent reflections	*l* = −14→14

### Refinement

**Table:**

Refinement on *F*^2^	Primary atom site location: structure-invariant direct methods
Least-squares matrix: full	Secondary atom site location: difference Fourier map
*R*[*F*^2^ > 2σ(*F*^2^)] = 0.049	Hydrogen site location: inferred from neighbouring sites
*wR*(*F*^2^) = 0.138	H-atom parameters constrained
*S* = 1.07	*w* = 1/[σ^2^(*F*_o_^2^) + (0.0713*P*)^2^ + 0.0224*P*] where *P* = (*F*_o_^2^ + 2*F*_c_^2^)/3
4508 reflections	(Δ/σ)_max_ < 0.001
272 parameters	Δρ_max_ = 0.16 e Å^−^^3^
0 restraints	Δρ_min_ = −0.14 e Å^−^^3^

### Special details

**Table:**

Geometry. All e.s.d.'s (except the e.s.d. in the dihedral angle between two l.s. planes) are estimated using the full covariance matrix. The cell e.s.d.'s are taken into account individually in the estimation of e.s.d.'s in distances, angles and torsion angles; correlations between e.s.d.'s in cell parameters are only used when they are defined by crystal symmetry. An approximate (isotropic) treatment of cell e.s.d.'s is used for estimating e.s.d.'s involving l.s. planes.
Refinement. Refinement of *F*^2^ against ALL reflections. The weighted *R*-factor *wR* and goodness of fit *S* are based on *F*^2^, conventional *R*-factors *R* are based on *F*, with *F* set to zero for negative *F*^2^. The threshold expression of *F*^2^ > σ(*F*^2^) is used only for calculating *R*-factors(gt) *etc*. and is not relevant to the choice of reflections for refinement. *R*-factors based on *F*^2^ are statistically about twice as large as those based on *F*, and *R*- factors based on ALL data will be even larger.

### Fractional atomic coordinates and isotropic or equivalent isotropic displacement parameters (Å^2^)

**Table:**

	*x*	*y*	*z*	*U*_iso_*/*U*_eq_	
C1	0.7361 (3)	−0.3823 (3)	0.2434 (4)	0.1697 (14)	
H1A	0.6724	−0.4471	0.2479	0.255*	
H1B	0.7775	−0.4104	0.3098	0.255*	
H1C	0.8142	−0.3665	0.1613	0.255*	
C2	0.6470 (3)	−0.2591 (2)	0.2611 (4)	0.1069 (8)	
C3	0.6537 (2)	−0.1587 (2)	0.1586 (3)	0.0959 (7)	
H3	0.7134	−0.1658	0.0765	0.115*	
C4	0.57379 (17)	−0.04748 (18)	0.17503 (17)	0.0716 (5)	
H4	0.5805	0.0185	0.1037	0.086*	
C5	0.48412 (15)	−0.03221 (14)	0.29525 (15)	0.0550 (4)	
C6	0.47896 (19)	−0.13296 (16)	0.39899 (18)	0.0721 (5)	
H6	0.4213	−0.1255	0.4815	0.086*	
C7	0.5588 (3)	−0.24457 (19)	0.3811 (3)	0.0993 (7)	
H7	0.5526	−0.3111	0.4519	0.119*	
C8	0.38850 (14)	0.08493 (13)	0.31928 (13)	0.0487 (3)	
H8	0.4107	0.1138	0.3847	0.058*	
C9	0.22214 (14)	0.05094 (13)	0.37594 (12)	0.0483 (3)	
H9	0.2076	−0.0218	0.4497	0.058*	
C10	0.12098 (15)	0.15808 (13)	0.42180 (13)	0.0510 (4)	
C11	0.12221 (18)	0.22548 (15)	0.50885 (14)	0.0651 (4)	
H11	0.1945	0.2096	0.5460	0.078*	
C12	0.0134 (2)	0.31740 (17)	0.53970 (17)	0.0785 (5)	
H12	0.0128	0.3636	0.5980	0.094*	
C13	−0.0940 (2)	0.34070 (16)	0.48454 (19)	0.0795 (6)	
H13	−0.1660	0.4026	0.5063	0.095*	
C14	−0.09647 (17)	0.27467 (15)	0.39865 (17)	0.0693 (5)	
H14	−0.1693	0.2912	0.3620	0.083*	
C15	0.01168 (15)	0.18229 (13)	0.36693 (14)	0.0543 (4)	
C16	0.03253 (15)	0.09317 (14)	0.28503 (13)	0.0544 (4)	
C17	0.15716 (14)	0.01666 (13)	0.28738 (13)	0.0498 (3)	
C18	0.19680 (16)	−0.07957 (15)	0.22107 (15)	0.0618 (4)	
H18	0.2802	−0.1298	0.2210	0.074*	
C19	0.1106 (2)	−0.09985 (19)	0.15508 (17)	0.0777 (5)	
H19	0.1366	−0.1639	0.1099	0.093*	
C20	−0.0136 (2)	−0.0261 (2)	0.15556 (17)	0.0827 (6)	
H20	−0.0713	−0.0423	0.1120	0.099*	
C21	−0.05361 (18)	0.07084 (18)	0.21919 (16)	0.0729 (5)	
H21	−0.1370	0.1207	0.2182	0.087*	
C22	0.41980 (15)	0.19632 (14)	0.20298 (13)	0.0547 (4)	
H22A	0.4240	0.1651	0.1298	0.066*	
H22B	0.3386	0.2571	0.2186	0.066*	
C23	0.56189 (15)	0.26443 (13)	0.17041 (14)	0.0538 (4)	
C24	0.63527 (15)	0.34181 (13)	0.03808 (14)	0.0538 (4)	
C25	0.76455 (17)	0.40522 (16)	0.01130 (17)	0.0735 (5)	
H25	0.8040	0.3976	0.0753	0.088*	
C26	0.8347 (2)	0.47915 (18)	−0.1090 (2)	0.0910 (6)	
H26	0.9210	0.5215	−0.1257	0.109*	
C27	0.7787 (2)	0.49087 (17)	−0.2040 (2)	0.0899 (7)	
H27	0.8269	0.5410	−0.2851	0.108*	
C28	0.6513 (2)	0.42882 (18)	−0.18013 (17)	0.0857 (6)	
H28	0.6133	0.4362	−0.2450	0.103*	
C29	0.57968 (18)	0.35505 (15)	−0.05867 (15)	0.0679 (4)	
H29	0.4927	0.3138	−0.0423	0.081*	
O1	0.61395 (13)	0.25998 (12)	0.25284 (11)	0.0812 (4)	

### Atomic displacement parameters (Å^2^)

**Table:**

	*U*^11^	*U*^22^	*U*^33^	*U*^12^	*U*^13^	*U*^23^
C1	0.148 (3)	0.126 (2)	0.296 (4)	0.073 (2)	−0.124 (3)	−0.114 (3)
C2	0.0798 (15)	0.0880 (16)	0.192 (3)	0.0286 (12)	−0.0758 (18)	−0.0689 (18)
C3	0.0586 (11)	0.1152 (18)	0.139 (2)	0.0149 (12)	−0.0379 (12)	−0.0747 (16)
C4	0.0486 (9)	0.0858 (12)	0.0836 (12)	−0.0001 (8)	−0.0194 (8)	−0.0312 (10)
C5	0.0415 (7)	0.0599 (9)	0.0671 (10)	−0.0025 (6)	−0.0243 (7)	−0.0124 (7)
C6	0.0647 (10)	0.0668 (11)	0.0898 (12)	0.0028 (8)	−0.0416 (9)	−0.0067 (9)
C7	0.0926 (15)	0.0659 (12)	0.160 (2)	0.0136 (11)	−0.0783 (16)	−0.0153 (14)
C8	0.0408 (7)	0.0543 (8)	0.0487 (8)	−0.0069 (6)	−0.0157 (6)	−0.0054 (6)
C9	0.0419 (7)	0.0492 (8)	0.0451 (7)	−0.0076 (6)	−0.0106 (6)	0.0002 (6)
C10	0.0441 (7)	0.0511 (8)	0.0455 (7)	−0.0101 (6)	−0.0036 (6)	−0.0038 (6)
C11	0.0632 (10)	0.0676 (10)	0.0542 (9)	−0.0122 (8)	−0.0078 (7)	−0.0108 (8)
C12	0.0831 (13)	0.0647 (11)	0.0676 (11)	−0.0099 (10)	0.0027 (10)	−0.0208 (9)
C13	0.0658 (11)	0.0590 (10)	0.0824 (12)	0.0019 (8)	0.0056 (10)	−0.0102 (9)
C14	0.0501 (9)	0.0601 (10)	0.0757 (11)	−0.0017 (7)	−0.0066 (8)	0.0002 (9)
C15	0.0416 (7)	0.0509 (8)	0.0541 (8)	−0.0063 (6)	−0.0060 (6)	0.0025 (7)
C16	0.0419 (8)	0.0611 (9)	0.0510 (8)	−0.0103 (7)	−0.0113 (6)	−0.0005 (7)
C17	0.0397 (7)	0.0519 (8)	0.0492 (8)	−0.0123 (6)	−0.0079 (6)	−0.0036 (6)
C18	0.0486 (8)	0.0663 (10)	0.0673 (10)	−0.0096 (7)	−0.0115 (7)	−0.0196 (8)
C19	0.0693 (11)	0.0951 (13)	0.0718 (11)	−0.0202 (10)	−0.0151 (9)	−0.0320 (10)
C20	0.0696 (12)	0.1190 (16)	0.0667 (11)	−0.0226 (11)	−0.0270 (9)	−0.0210 (11)
C21	0.0524 (9)	0.0937 (13)	0.0696 (10)	−0.0061 (9)	−0.0252 (8)	−0.0058 (10)
C22	0.0415 (7)	0.0595 (9)	0.0554 (8)	−0.0073 (6)	−0.0142 (6)	−0.0012 (7)
C23	0.0434 (8)	0.0534 (8)	0.0593 (9)	−0.0042 (6)	−0.0130 (7)	−0.0098 (7)
C24	0.0447 (8)	0.0457 (8)	0.0595 (9)	−0.0032 (6)	−0.0040 (7)	−0.0129 (7)
C25	0.0574 (9)	0.0658 (10)	0.0840 (12)	−0.0167 (8)	−0.0055 (8)	−0.0170 (9)
C26	0.0678 (11)	0.0761 (13)	0.0967 (15)	−0.0269 (9)	0.0106 (11)	−0.0130 (11)
C27	0.0867 (14)	0.0625 (11)	0.0737 (12)	−0.0086 (10)	0.0195 (11)	−0.0025 (9)
C28	0.0953 (14)	0.0782 (12)	0.0606 (10)	−0.0041 (11)	−0.0087 (10)	−0.0026 (9)
C29	0.0639 (10)	0.0646 (10)	0.0596 (10)	−0.0111 (8)	−0.0076 (8)	−0.0043 (8)
O1	0.0721 (8)	0.0992 (9)	0.0744 (8)	−0.0310 (7)	−0.0316 (6)	−0.0031 (7)

### Geometric parameters (Å, º)

**Table:**

C1—C2	1.532 (3)	C14—C15	1.390 (2)
C1—H1A	0.9600	C14—H14	0.9300
C1—H1B	0.9600	C15—C16	1.462 (2)
C1—H1C	0.9600	C16—C21	1.388 (2)
C2—C7	1.374 (3)	C16—C17	1.4026 (19)
C2—C3	1.375 (3)	C17—C18	1.386 (2)
C3—C4	1.382 (3)	C18—C19	1.382 (2)
C3—H3	0.9300	C18—H18	0.9300
C4—C5	1.382 (2)	C19—C20	1.378 (3)
C4—H4	0.9300	C19—H19	0.9300
C5—C6	1.390 (2)	C20—C21	1.373 (3)
C5—C8	1.5171 (19)	C20—H20	0.9300
C6—C7	1.388 (3)	C21—H21	0.9300
C6—H6	0.9300	C22—C23	1.5091 (19)
C7—H7	0.9300	C22—H22A	0.9700
C8—C22	1.5186 (18)	C22—H22B	0.9700
C8—C9	1.5510 (18)	C23—O1	1.2167 (17)
C8—H8	0.9800	C23—C24	1.490 (2)
C9—C17	1.5099 (19)	C24—C29	1.377 (2)
C9—C10	1.5141 (19)	C24—C25	1.388 (2)
C9—H9	0.9800	C25—C26	1.375 (2)
C10—C11	1.383 (2)	C25—H25	0.9300
C10—C15	1.395 (2)	C26—C27	1.363 (3)
C11—C12	1.389 (2)	C26—H26	0.9300
C11—H11	0.9300	C27—C28	1.373 (3)
C12—C13	1.380 (3)	C27—H27	0.9300
C12—H12	0.9300	C28—C29	1.386 (2)
C13—C14	1.365 (3)	C28—H28	0.9300
C13—H13	0.9300	C29—H29	0.9300
			
C2—C1—H1A	109.5	C15—C14—H14	120.6
C2—C1—H1B	109.5	C14—C15—C10	120.40 (15)
H1A—C1—H1B	109.5	C14—C15—C16	131.16 (15)
C2—C1—H1C	109.5	C10—C15—C16	108.37 (13)
H1A—C1—H1C	109.5	C21—C16—C17	120.27 (15)
H1B—C1—H1C	109.5	C21—C16—C15	130.73 (15)
C7—C2—C3	117.6 (2)	C17—C16—C15	108.82 (13)
C7—C2—C1	120.8 (3)	C18—C17—C16	119.94 (14)
C3—C2—C1	121.6 (3)	C18—C17—C9	129.88 (13)
C2—C3—C4	121.5 (2)	C16—C17—C9	110.01 (12)
C2—C3—H3	119.3	C19—C18—C17	119.05 (15)
C4—C3—H3	119.3	C19—C18—H18	120.5
C3—C4—C5	121.37 (19)	C17—C18—H18	120.5
C3—C4—H4	119.3	C20—C19—C18	120.66 (17)
C5—C4—H4	119.3	C20—C19—H19	119.7
C4—C5—C6	117.19 (15)	C18—C19—H19	119.7
C4—C5—C8	124.00 (14)	C21—C20—C19	121.19 (16)
C6—C5—C8	118.78 (14)	C21—C20—H20	119.4
C7—C6—C5	120.8 (2)	C19—C20—H20	119.4
C7—C6—H6	119.6	C20—C21—C16	118.86 (16)
C5—C6—H6	119.6	C20—C21—H21	120.6
C2—C7—C6	121.5 (2)	C16—C21—H21	120.6
C2—C7—H7	119.2	C23—C22—C8	113.76 (11)
C6—C7—H7	119.2	C23—C22—H22A	108.8
C5—C8—C22	114.16 (12)	C8—C22—H22A	108.8
C5—C8—C9	111.08 (11)	C23—C22—H22B	108.8
C22—C8—C9	110.72 (10)	C8—C22—H22B	108.8
C5—C8—H8	106.8	H22A—C22—H22B	107.7
C22—C8—H8	106.8	O1—C23—C24	119.90 (13)
C9—C8—H8	106.8	O1—C23—C22	120.24 (13)
C17—C9—C10	102.26 (11)	C24—C23—C22	119.81 (13)
C17—C9—C8	116.84 (11)	C29—C24—C25	118.31 (14)
C10—C9—C8	114.00 (11)	C29—C24—C23	123.07 (13)
C17—C9—H9	107.8	C25—C24—C23	118.61 (15)
C10—C9—H9	107.8	C26—C25—C24	120.53 (18)
C8—C9—H9	107.8	C26—C25—H25	119.7
C11—C10—C15	120.21 (14)	C24—C25—H25	119.7
C11—C10—C9	129.23 (14)	C27—C26—C25	120.48 (18)
C15—C10—C9	110.51 (13)	C27—C26—H26	119.8
C10—C11—C12	118.74 (17)	C25—C26—H26	119.8
C10—C11—H11	120.6	C26—C27—C28	120.15 (17)
C12—C11—H11	120.6	C26—C27—H27	119.9
C13—C12—C11	120.51 (17)	C28—C27—H27	119.9
C13—C12—H12	119.7	C27—C28—C29	119.50 (19)
C11—C12—H12	119.7	C27—C28—H28	120.3
C14—C13—C12	121.29 (17)	C29—C28—H28	120.3
C14—C13—H13	119.4	C24—C29—C28	121.02 (16)
C12—C13—H13	119.4	C24—C29—H29	119.5
C13—C14—C15	118.86 (17)	C28—C29—H29	119.5
C13—C14—H14	120.6		
			
C7—C2—C3—C4	−0.7 (3)	C10—C15—C16—C21	173.55 (14)
C1—C2—C3—C4	−179.65 (18)	C14—C15—C16—C17	−178.32 (14)
C2—C3—C4—C5	0.3 (3)	C10—C15—C16—C17	−1.52 (15)
C3—C4—C5—C6	0.6 (2)	C21—C16—C17—C18	1.7 (2)
C3—C4—C5—C8	−177.22 (14)	C15—C16—C17—C18	177.33 (12)
C4—C5—C6—C7	−1.2 (2)	C21—C16—C17—C9	−174.09 (12)
C8—C5—C6—C7	176.79 (14)	C15—C16—C17—C9	1.59 (14)
C3—C2—C7—C6	0.1 (3)	C10—C9—C17—C18	−176.22 (13)
C1—C2—C7—C6	179.10 (19)	C8—C9—C17—C18	58.58 (18)
C5—C6—C7—C2	0.8 (3)	C10—C9—C17—C16	−1.03 (13)
C4—C5—C8—C22	−10.92 (19)	C8—C9—C17—C16	−126.24 (12)
C6—C5—C8—C22	171.25 (12)	C16—C17—C18—C19	−1.1 (2)
C4—C5—C8—C9	115.14 (15)	C9—C17—C18—C19	173.70 (13)
C6—C5—C8—C9	−62.69 (16)	C17—C18—C19—C20	−0.4 (2)
C5—C8—C9—C17	−70.05 (15)	C18—C19—C20—C21	1.3 (3)
C22—C8—C9—C17	57.89 (16)	C19—C20—C21—C16	−0.7 (3)
C5—C8—C9—C10	170.88 (11)	C17—C16—C21—C20	−0.8 (2)
C22—C8—C9—C10	−61.18 (15)	C15—C16—C21—C20	−175.36 (14)
C17—C9—C10—C11	177.56 (13)	C5—C8—C22—C23	−73.97 (16)
C8—C9—C10—C11	−55.39 (18)	C9—C8—C22—C23	159.78 (12)
C17—C9—C10—C15	0.08 (13)	C8—C22—C23—O1	−24.5 (2)
C8—C9—C10—C15	127.14 (12)	C8—C22—C23—C24	158.07 (12)
C15—C10—C11—C12	−0.3 (2)	O1—C23—C24—C29	−177.53 (14)
C9—C10—C11—C12	−177.51 (13)	C22—C23—C24—C29	−0.1 (2)
C10—C11—C12—C13	0.1 (2)	O1—C23—C24—C25	1.5 (2)
C11—C12—C13—C14	0.0 (3)	C22—C23—C24—C25	178.93 (13)
C12—C13—C14—C15	0.1 (2)	C29—C24—C25—C26	0.0 (2)
C13—C14—C15—C10	−0.2 (2)	C23—C24—C25—C26	−179.05 (14)
C13—C14—C15—C16	176.26 (14)	C24—C25—C26—C27	−0.3 (3)
C11—C10—C15—C14	0.3 (2)	C25—C26—C27—C28	0.1 (3)
C9—C10—C15—C14	178.05 (12)	C26—C27—C28—C29	0.4 (3)
C11—C10—C15—C16	−176.89 (11)	C25—C24—C29—C28	0.5 (2)
C9—C10—C15—C16	0.85 (15)	C23—C24—C29—C28	179.53 (15)
C14—C15—C16—C21	−3.2 (3)	C27—C28—C29—C24	−0.8 (3)

### Hydrogen-bond geometry (Å, º)

Cg is the centroid of the C10–C15 ring.

**Table:**

*D*—H···*A*	*D*—H	H···*A*	*D*···*A*	*D*—H···*A*
C27—H27···*Cg*^i^	0.93	2.76	3.617 (2)	154

Symmetry code: (i) −*x*+1, −*y*+1, −*z*.
